# Comfort levels in discussing tobacco smoking among hospital staff in a children’s hospital

**DOI:** 10.18332/tpc/162438

**Published:** 2023-06-01

**Authors:** Yannan Li, Cordelia Eliaho, Bian Liu, Karen Wilson

**Affiliations:** 1 Department of Environmental Medicine and Public Health, Icahn School of Medicine at Mount Sinai, New York, United States; 2 Medical College of Wisconsin, Milwaukee, United States; 3 Department of Population Health Science and Policy, Icahn School of Medicine at Mount Sinai, New York, United States; 4 Department of Pediatrics, University of Rochester School of Medicine and Dentistry, Rochester, United States

**Keywords:** smoking, tobacco control, hospital, perceptions, environmental health

## Abstract

**INTRODUCTION:**

Hospital staff discussing smoking with children and their families can impact tobacco control, which is crucial in reducing the harmful effects of tobacco smoke exposure. Our study aims to assess staff comfort level in discussing smoking with patients or their families, and coworkers, after the implementation of a hospital-wide tobacco control policy.

**METHODS:**

This cross-sectional study included 2340 staff members who completed an anonymous online survey in a large urban children’s hospital in 2019. The main outcomes of interest were the comfort level in discussing smoking with patients or their families, and co-workers. We used multivariable logistic regression to identify whether the comfort level varied by sex, age, job type, and smoking status.

**RESULTS:**

Most of the respondents (83.8%) were female, 41.2% were aged 18–35 years, 57.6% worked as clinical staff, and 15.5% were ever smokers. Compared to males, females were less likely to feel very comfortable in asking patients or their families about their smoking tobacco (adjusted odds ratio, AOR=0.72; 95% CI: 0.56–0.92) or talking to co-workers about the health risks associated with their smoking (AOR=0.71; 95% CI: 0.54–0.93). Staff who were non-smokers were less likely to feel very comfortable in talking to co-workers about the health risks associated with their smoking (AOR=0.60; 95% CI: 0.45–0.78). The odds of feeling very comfortable in discussing smoking were consistently lower among those aged 18–35 years than their older counterparts. Clinical staff were more likely than non-clinical staff to feel very comfortable in discussing with patients and their parents about smoking, but there was no difference when talking to co-workers.

**CONCLUSIONS:**

We found differences in staff comfort level in discussing smoking with patients or their families, and coworkers, by sex, age, job type, and smoking status. These results can guide training and identify potential barriers and improve tailored tobacco control training programs and policies for hospital staff.

## INTRODUCTION

The detrimental effects of tobacco smoke on the human body, especially for children, have been well-documented^1-3^. Parental smoking is a significant source of secondhand smoke among children, and an elevated risk of initiating smoking by children growing up^4,5^. Tobacco control and prevention are essential to reduce tobacco smoke exposure and the risk of adverse health effects both in children and adults^6^. Increasing workplace tobacco control policies and utilizing the 5As framework and the ‘Making Every Contact Count’ (MECC) approach, have contributed to more negative attitudes about smoking and resulted in a decrease in secondhand smoke exposure^7-9^. Studies based on nationally representative samples found that indoor workers who self-reported a 100% smoke-free policy at work, were less likely to smoke combustible tobacco and were more likely to quit smoking^10,11^. The hospitalization of a child is an important opportunity to practice tobacco control, and to include evidence-based cessation programs for healthcare providers, supporting staff, and parents. However, it has been demonstrated in the literature that not all hospital staff members are equally capable of providing high-quality tobacco control services to patients. For example, a recent cross-sectional study conducted in Wales (UK) found that female staff members and younger professionals were less likely to report feeling comfortable initiating health behavior conversations, including discussions about smoking cessation, across all topics^12^. A systematic review focused on nurses found no differences in the delivery of smoking cessation interventions based on the nurses’ smoking status^13^.

The level of comfort in discussing smoking for hospital staff could potentially contribute to the success in reducing tobacco smoking and secondhand smoke exposure for patients, their parents, and workers in the children’s hospital; some demographic characteristics such as sex, age, job type, and smoking status might potentially influence the level of comfort in such discussions. Our study assessed the comfort level in discussing smoking with patients or their families, and co-workers, and examined potential differences by these subgroups. These findings will help improve tobacco control related training among pediatric hospital staff in the future and warrant future analysis in identifying potential gaps and barriers in implementing tobacco control policies within the hospital setting.

## METHODS

### Data source and study population

We conducted a cross-sectional study using an anonymous online survey at the Children’s Hospital, Colorado, in March 2019. Emails were sent out to 6821 individuals through three different blasts to a staff listserv and 2813 staff (41.2%) responded. After excluding 473 incomplete surveys, we included 2340 completed survey data for the final analysis. We used SurveyMonkey software (surveymonkey.com, San Mateo, CA) and Research Electronic Data Capture (REDCap) to collect data.

The current survey was modeled after a previous study within the same hospital^14^. There has been a randomized controlled trial (RCT) on parent smoking cessation intervention (INSPIRE)^15^. As a part of the RCT, we engaged hospital staff over 4 years to increase awareness of the importance of addressing tobacco smoke exposure. A survey on staff knowledge and perception of tobacco control and prevention policy was conducted at the end and six months after the end of the RCT in 2018, and the results from this earlier survey were previously published^15^.

### Survey questions and measures

The survey was designed to assess the reach of the hospital-wide tobacco control policy and related programs; prior to this survey, original questions were piloted with providers and staff at the same hospital at different time points. This is the latest assessment using this survey^15^. The survey consisted of questions from three domains. The first domain of questions collected demographic characteristics and information about job type and smoking status. The second domain of questions assessed the awareness and support of the hospital’s tobacco control policy and smoking cessation programs available to parents and hospital staff. The last domain consisted of three questions assessing staff level of comfort in discussing smoking with others.

The main outcomes of interest were the comfort level in discussing smoking with patients or families, and co-workers, based on the following three questions: 1) ‘How comfortable do you feel asking patients or their families about their smoking tobacco?’; 2) ‘How comfortable are you educating patients or their families about the health risks associated with their tobacco smoking?’; and 3) ‘How comfortable do you feel talking to co-workers about the health risks associated with their tobacco smoking?’. Participants could choose from ‘very comfortable’, ‘somewhat’, ‘a little’ and ‘not at all’ as responses. We dichotomized the responses as ‘very comfortable’ versus ‘not very comfortable’ (which included also ‘somewhat’, ‘a little’, and ‘not at all’) for each of the questions.

We considered the following four covariates, including age (18–35, 36–45, and >45 years), sex, job type, and smoking status. We grouped professions into two job types: clinical and non-clinical, where the former included nurses, respiratory therapists, advanced providers, attending physicians, fellow physicians, and resident physicians; the latter included social workers, case management staff, environmental service staff, food and nutrition service staff, and administrative staff. Ever smoker was self-defined as smoking at least 100 cigarettes in their lifetime, and current smokers were self-defined as currently smoking cigarettes every day.

### Statistical analysis

We summarized the comfort level in smoking discussion using frequency and proportions for the overall sample and by subgroups stratified on sex, age, job type, and smoking status. We then used multivariable logistic regression to identify whether any of the four covariates were statistically significantly associated with the comfort level and reported the adjusted odds ratios (AORs) and 95% confidence intervals (CIs) by adjusting for all other covariates. We set the statistical significance level at p<0.05. The analysis was conducted using SAS OnDemand for Academics (SAS Institute Inc, Cary, NC).

## RESULTS

Table 1 shows the characteristics of the overall study population of 2340 participants. Of the respondents without missing data: 1942 (83.8%) were female staff and 374 (16.2%) were male staff; staff aged 18–35 years represented 41.2%; clinical staff represented 57.6%; and there were 362 (15.5%) staff who self-reported as having smoked 100 cigarettes in their lifetime. Overall, of the total sample of 2340 participants, 33 (1.4%) currently smoked cigarettes every day.

**Table 1:** Demographic characteristics of the study population (N=2340)

*Characteristics*	*Categories*	*n*	*%*
**Gender**	Female	1942	83.85
	Male	374	16.15
	Missing (24)		
**Age** (years)	18–35	960	41.18
	36–45	686	29.43
	≥46	685	29.39
	Missing (9)		
**Job type**	Clinical staff	1259	57.59
	Non-clinical staff	927	42.41
	Missing (154)		
**Have you smoked at least 100 cigarettes in your lifetime?**	Don’t know or not sure	21	0.9
	No	1949	83.58
	Yes	362	15.52
	Missing (8)		
**Do you currently smoke cigarettes every day?**	No	341	90.89
	Yes	33	9.11
	Missing (17)		

Table 2 shows the awareness and support of smoking cessation programs for hospital staff and patients’ parents. Over 95% of the respondents agreed that the hospital should provide tobacco control related programs to staff, while the percentage supporting such programs for patients’ parents was over 85%. About two-thirds of the respondents were aware that the hospital had programs helping staff to quit smoking tobacco, where 41.8% of the respondents were aware of such a program for parents.

**Table 2:** Staff awareness of and support for smoking cessation programs available in the hospital for parents and staff (N=2340)

*Question*	*Response*	*n*	*%*
Are you aware of any programs at CHC to help parents quit smoking tobacco?	No	1354	58.2
	Yes	971	41.8
Do you think that CHC should provide support to parents who want to quit smoking tobacco?	No	94	4
	Yes	1987	85.3
	Don't know/not sure	248	10.7
Are you aware of any programs at CHC to help staff quit smoking tobacco?	No	801	34.6
	Yes	1517	65.4
Do you think CHC should provide any support to staff who want to quit smoking tobacco?	No	35	1.5
	Yes	2229	95.9
	Don't know/not sure	61	2.6

CHC: Children’s Hospital Colorado.

We found that the level of comfort in talking about smoking differed by sex, age, job type, and smoking status, and the difference varied by the topics of the discussion and with whom were the discussions, adjusting for all other variables (Table 3). Overall, 41.4% of the respondents felt very comfortable asking patients or their families about their smoking status, 25.8% felt very comfortable educating patients/families about the health risks of their smoking tobacco, and 19.5% felt very comfortable talking to co-workers about the health risks associated with their tobacco smoking. Compared to males, females were less likely to feel very comfortable in asking patients or their families about their smoking tobacco (AOR=0.72; 95% CI: 0.56–0.92) or talking to co-workers about the health risk associated with their tobacco smoking (AOR=0.71; 95% CI: 0.54–0.93). The youngest age group (18–35 years) was the least comfortable in all three outcomes regarding the discussion on tobacco smoking (Table 3). The proportion of those who felt very comfortable in discussing smoking was higher among clinical staff than non-clinical staff. Clinical staff were also more likely to feel very comfortable in asking patients/families about their smoking tobacco (AOR=2.81; 95% CI: 2.34–3.39), and educating patients/families about the health risk of tobacco smoking (AOR=1.99; 95% CI: 1.61– 2.45), while the association was not statistically significant for talking to co-workers about the health risks of tobacco smoking (AOR=1.03; 95% CI: 0.83–1.29). Compared to ever and current smokers, non-smokers were less likely to feel ‘very comfortable’ talking to co-workers about smoking (AOR=0.60; 95% CI: 0.45–0.78) (Table 3).

**Table 3:** Staff level of comfort in discussing smoking and prevention with patients, families, and co-workers, stratified by gender, age, job type, and smoking status*

*Variable*		*How comfortable do you feel asking patients or their families about their smoking tobacco?*			*How comfortable are you educating patients or their families about the health risks associated with their tobacco smoking?*			*How comfortable do you feel talking to co-workers about the health risks associated with their tobacco smoking?*		
	*n*	*Very %*	*AOR (95% CI)*	*p*	*Very %*	*AOR (95% CI)*	*p*	*Very %*	*AOR (95% CI)*	*p*
**Overall**		41.4	-	-	25.8	-	-	19.5	-	-
**Gender**										
Female	1942	40.4	0.72 (0.56–0.92)	0.0073	25.1	0.83 (0.64–1.08)	0.1630	18.5	0.71 (0.54–0.93)	0.0141
Male (Ref.)	374	46.9	1		29.7	1		24.9	1	
**Age** (years)										
18–35	960	37.7	0.74 (0.59–0.92)	0.0012	19.5	0.47 (0.37–0.60)	<0.0001	16.9	0.72 (0.56–0.94)	0.0767
36–45	686	45.4	1.01 (0.80–1.27)	0.1085	29.1	0.83 (0.65–1.05)	0.0851	19.7	0.79 (0.60–1.03)	0.5246
≥46 (Ref.)	685	42.4	1		31.3	1		23.1	1	
**Job type**										
Clinical	1259	52.2	2.81 (2.34–3.39)	<0.0001	31.3	1.99 (1.61–2.45)	<0.0001	19.3	1.03 (0.83–1.29)	0.7634
Non-clinical (Ref.)	927	28.7	1		19.7	1		20.0	1	
**Smoking status**										
Never smoker	1949	40.7	0.89 (0.69–1.14)	0.5565	25.1	0.88 (0.67–1.15)	0.6074	17.8	0.60 (0.45–0.78)	0.0009
Ever or current smoker (Ref.)	362	45.1	1		29.9	1		28.3	1	

* The results have been adjusted for all other covariates. For example, when assessing the effect of gender, the model adjusted for age, job type, and smoking status.

## DISCUSSION

We utilized a cross-sectional survey to assess staff comfort level in discussing smoking with patients and their families, as well as co-workers, in a large urban children’s hospital. We found that differences existed in the comfort levels of discussing smoking with patients/families and co-workers by sex, age, job type, and smoking status. The results may help identify barriers for discussing smoking and improve tailored tobacco control training for hospital staff.

Our study results indicated a notable predominance of female respondents, which is consistent with the sex distribution of the hospital workforce in the United States. As of 2022, women hold 75% of total employment in hospitals^16^. The high proportion (over 80%) of female respondents in our study may also be attributed to the increasing percentage of women in the healthcare workforce, particularly in pediatric, obstetrics and gynecology, child and adolescent psychiatry, and neonatal–perinatal medicine specialties^17^. Moreover, female staff might potentially have more concerns about tobacco control, but it is unknown how largely this effect would be. Additionally, the age distribution observed in our study was also in line with the national trend, with a median age range that matched the reported range (35–45 years) for the overall hospital workforce in the US^18^.

The age and sex differences we found were consistent with previous studies. As a previous study from the same hospital indicated, older male general physicians were more likely to initiate smoking cessation conversation with their patients^19^. This finding suggests that younger and female staff and providers may have barriers to discussing smoking with others; these barriers may have to do with dynamics of gender and age. To address this, a study provided evidence on using special education content on smoking cessation to boost the comfort level of behavioral counselling in bedside nurses^20^. Such a finding suggested that the management of the hospital should provide support and specialized education content regarding tobacco control to our female and younger staff. Future studies to identify such barriers and implement targeted interventions are thus warranted.

Our study showed that about 57% of the hospital staff were working clinically, which was also consistent with the national data^21^. When looking into the differences between clinical and non-clinical staff, we found that non-clinical staff were less comfortable in discussing smoking. This is not surprising, as they may in general lack specific training in asking patients/families about their smoking and providing advice on tobacco control and prevention^22,23^. The proportion of respondents who were highly aware of the available smoking cessation programs in the hospital to parents/families were approximately 30% lower than that reported in the previous survey, which was limited to clinicians. The inclusion of non-clinical staff in the current survey may help explain some of the discrepancies. The current results suggest non-clinical staff could be an important group to be included in a wider campaign to increase staff knowledge and confidence in tobacco control and prevention policy. Future tobacco control training should have a particular focus on non-clinical staff.

About 9% of our respondents reported ever smoking, which is between the reported smoking prevalence from a national data studying healthcare professionals and hospital workers^24,25^. Interestingly, hospital staff who identified as ever smokers were more comfortable discussing smoking in all 3 questions. Non-smokers tend to be younger than smokers, and ever smokers might have more previous or current interactions with tobacco that help them proceed with more confidence, compared to non-smokers. However, limited literature was found on this topic. Since most of the respondents were non-smokers, continuing to provide education to support knowledge about tobacco will be critical.

The current analysis revealed a significant level of cognizance and endorsement for tobacco control policies among hospital staff, but relatively low awareness of the existing tobacco cessation programs either targeting hospital employees or patient’s parents. To enhance the effectiveness of tobacco control policies, efforts should be directed towards increasing awareness and improving utilization rates of such programs.

There is a need to provide appropriate support and bolster confidence, especially for younger, female, and non-clinical hospital staff, in strategies of initiating conversations about tobacco control with patients, which could be generalized from other topics such as palliative care and learn to target patient concern based on their objections^26,27^. A culture of teamwork and collaboration among staff may also be helpful, for example, pairing with older male clinicians may also be an effective strategy to improve communication and confidence^28^. To target younger staff, an innovative approach such as in the context of age-specific training programs or web-based educational tools, might be more effective than traditional meetings or training approaches. Additionally, providing ongoing feedback and support to these staff members may be effective in reinforcing and improving their skills over time.

### Strengths and limitations

The current study has a much larger sample size and includes staff from a variety of job types and age groups than the prior study. By comparing our study population to national statistics, we have determined that it is well-represented and has minimized the potential for selection bias. However, this study has a few limitations. As this is a cross-sectional study design and based on self-reported responses, we are limited in making causal inferences and our findings are subject to recall and information bias. No longitudinal linkage between this and the previous study was available, due to the anonymous nature of the survey. Finally, given that our study sample consisted primarily of staff members who were more inclined towards healthy lifestyles and associated behaviors (such as low smoking rates), it is important to exercise caution when interpreting the findings.

## CONCLUSIONS

Strong perceptions of tobacco control policies and awareness of the smoking cessation programs for staff and patients’ parents were found in this hospital after the implementation of a tobacco cessation program for parents and educational interventions within the hospital. As we seek to have more effective tobacco control and innovative ways to discuss smoking cessation, understanding the disparities in different subgroups of hospital staff is critical to bridging gaps. Our study suggests that female staff, younger staff, non-clinical staff, and staff who were non-smokers may benefit from studies identifying barriers and using targeted programs to boost their comfort level in discussing smoking to further protect patients/families and create a healthier work environment.

## Data Availability

The data supporting this research cannot be made available for privacy or other reasons.
